# Associations Between the Severity of Obstructive Sleep Apnea and Arterial Stiffness Estimated by Pulse Wave Velocity Assessment: A Cross‐Sectional Study

**DOI:** 10.1002/hsr2.71861

**Published:** 2026-03-11

**Authors:** Erica Silva, Jessica Giovana Teixeira de Andrade, Eduardo Rodrigues Ferreira Gomes de Camargos, Olivia Mendonça Nunes, Gabrielle Santos Pontello Neves, Patrícia de Souza Pereira, Jose Felippe Pinho da Silva, Daniel Mendes‐Pinto, Maria da Glória Rodrigues‐Machado, Bruno Almeida Rezende

**Affiliations:** ^1^ Faculdade de Ciências Médicas de Minas Gerais Belo Horizonte Brazil; ^2^ Hospital da Polícia Militar de Minas Gerais Belo Horizonte Brazil; ^3^ Hospital Felício Rocho Belo Horizonte Brazil

**Keywords:** arterial stiffness, cardiovascular risk, obstructive sleep apnea, polysomnography, pulse wave velocity

## Abstract

**Background and Aims:**

Obstructive sleep apnea (OSA) is a prevalent sleep disorder linked to cardiovascular diseases and is highly prevalent, especially in the elderly population. This study aimed to investigate the association between OSA severity and arterial stiffness, as measured by pulse wave velocity (PWV), in patients with moderate to severe OSA.

**Methods:**

This cross‐sectional study included 60 adults with moderate‐to‐severe OSA, confirmed by polysomnography (PSG). Arterial stiffness was measured by brachial artery oscillometry. Polysomnographic parameters, including the apnea–hypopnea index (AHI), minimum oxygen saturation (SpO2), and sleep efficiency, were analyzed to determine their correlation with PWV. Correlations between PWV and PSG parameters were performed using Spearman's correlation coefficient. Additionally, multivariate regression analysis was conducted to adjust for confounders, including age, sex, BMI, hypertension, diabetes, and medication use.

**Results:**

The mean age of participants was 62.07 ± 8.83 years, with 55% being male. A significant positive correlation was found between PWV and AHI (*r* = 0.4724; 95% CI = 0.2438 to 0.6512; *p* < 0.001), indicating that arterial stiffness increases with OSA severity. Additionally, an inverse relationship was observed between PWV and minimum SpO_2_ (*r* = ‐0.2995; 95% CI = –0.5234 to –0.03684; *p* = 0.02), as well as between PWV and sleep efficiency (*r* = –0.2702; 95% CI = −0.4999 to –0.004971; *p* = 0.04). However, when adjusted for age and other potential confounders using multivariate regression, these associations between PSG parameters and arterial stiffness lost statistical significance. Age emerged as the primary independent predictor of PWV in this population.

**Conclusion:**

The study confirms a significant initial association between the severity of OSA, as indicated by AHI and minimum SpO_2_, and arterial stiffness. The subsequent analysis highlights that the relationship between sleep parameters and PWV is primarily age‐dependent, emphasizing the importance of rigorous cardiovascular risk assessment in aging OSA patients.

## 1 Introduction

Obstructive sleep apnea (OSA) is a prevalent sleep disorder characterized by repeated episodes of partial or complete obstruction of the upper airway during sleep, causing intermittent hypoxia. This causes breathing to stop (apnea) or become very shallow (hypopnea) for short periods, often resulting in snoring, gasping, fragmented sleep, and daytime fatigue. Once clinical examination and patient history raise suspicion of OSA, a full‐night polysomnography (PSG) must be conducted to accurately classify the severity of the condition [1]. This classification is based on the number of respiratory events, including apneas and hypopneas, occurring per hour of sleep, quantified by the Apnea–Hypopnea Index (AHI) [2]. An AHI greater than 15 indicates moderate apnea, while values above 30 represent severe apnea. In addition to AHI, other parameters assessed in PSG are very useful in assessing the severity of the condition, such as average and minimum oxygen saturation (SpO_2_) and saturation time ˂ 90% (T/SpO_2_ < 90%) [2].

OSA is an independent risk factor for cardiovascular morbidity and mortality [3, 4]. It is believed that intermittent hypoxia, activation of the sympathetic nervous system, and negative intrathoracic pressure resulting from OSA can cause vascular and cardiac morphofunctional changes that significantly increase the predisposition to arterial hypertension, coronary artery disease, heart failure, arrhythmias, and stroke [3, 5, 6]. Although we currently have efficient therapy for the treatment of OSA, mainly through the use of continuous positive airway pressure (CPAP), there is still no consensus in the literature that the treatment of OSA reduces cardiovascular risk, since recent clinical studies did not indicate a reduction in cardiovascular risk from the treatment of OSA [7, 8, 9, 10]. Other authors believe that to observe an improvement in cardiovascular risk, therapy for the condition must be sustained over an extended period [11, 12].

Due to the close relationship between OSA and cardiovascular diseases, adequate cardiovascular risk stratification in patients with OSA is necessary, especially in those who have more severe conditions. New cardiovascular disease markers, such as pulse wave velocity (PWV), Augmentation index (Aix), and Augmentation Pressure (AP), have been proposed [13, 14]. These parameters are indicative of vascular aging and are regarded as the main indicators of arterial stiffness, an independent predictor of future cardiovascular events and mortality [15, 16]. These indicators can be indirectly accessed using arterial tonometry [17] or devices that estimate arterial stiffness by brachial artery oscillometry [18]. Even though arterial stiffness is considered intrinsic to the aging process, it is believed that it can also be influenced by factors and diseases such as OSA [17, 19]. The correlation between PWV and OSA severity has not yet been scientifically confirmed [17, 20, 21]. Furthermore, the coexistence of OSA and conventional risk factors such as hypertension, diabetes, hyperlipidemia, and obesity contributes to increased arterial stiffness, making it difficult to establish a causal relationship [22].

Herein, this study aimed to determine whether the main measures of OSA severity obtained by PSG were associated with arterial stiffness indices in patients with moderate to severe OSA.

## 2 Methods

### 2.1 Participants

This cross‐sectional study involved 60 adults enrolled in the Pulmonology outpatient clinic of the Hospital Militar de Minas Gerais (HPM‐MG). All patients seen at the center between March 2023 and November 2024 and met the study inclusion criteria were invited to participate.

Inclusion criteria included a convenience sample of patients of both sexes, aged 50 years and over, and diagnosed with moderate to severe OSA previously confirmed by type 1 PSG obtained within the last 90 days. None of the participants had received any treatment for OSA on the occasion.

Exclusion criteria included the presence of other sleep disorders, neuromuscular diseases, chronic obstructive pulmonary disease, cerebrovascular disease, renal failure, peripheral arterial disease, severe cognitive disorders, recent upper airway surgery, use of alternative treatments for OSA, and unavailability of PSG results and smokers. Patients with uncontrolled hypertension, defined as blood pressure levels exceeding the limits established by the Brazilian Guidelines of Hypertension (2020) for adequate blood pressure control, were excluded from the study [23]. Similarly, diabetic patients with glycemic levels indicative of poor blood pressure control, as per the American Diabetes Association, were also excluded [24]. These conditions significantly impact arterial stiffness and could introduce important confounding factors in the association between polysomnographic indices and arterial stiffness.

The variables investigated included anthropometric data, associated comorbidities, arterial stiffness indices, and PSG data. Information on anthropometric variables, clinical history, and clinical examination was gathered through anamnesis using a standardized collection form. Following the collection of anthropometric and clinical data, hemodynamic and vascular parameters, as well as arterial stiffness indices, were assessed. All vascular and hemodynamic assessments were performed in the morning. This study was approved by the Research Ethics Committee of the Faculdade de Ciências Médicas de Minas Gerais (approval report number: 5513761) and was conducted in accordance with the principles of the Declaration of Helsinki. All participants were informed of the research objectives and procedures beforehand and provided written consent by signing the consent form.

### 2.2 Ethics Approval

This study was performed in line with the principles of the Declaration of Helsinki. Approval was granted by the Research Ethics Committee of the Faculty of Medical Sciences of Minas Gerais (approval report n. 5513761).

### 2.3 Consent to Participate

All participants provided written informed consent after receiving full orientation regarding the potential risks and benefits of the research.

### 2.4 PSG Data

All participants had a recent diagnosis of OSA (within 3 months before evaluation) confirmed by baseline PSG, which was conducted in a sleep laboratory and observed by a professional present during data collection to oversee the extraction of the variables of interest [1].

The following polysomnographic data were considered for analysis: AHI, sleep efficiency, average and minimum oxygen saturation (SpO_2_), saturation time < 90%, awakening, phases of sleep, and sleep‐phase distribution (N1, N2, N3, and rapid eye movement or REM) [2]. Polysomnographic recordings were scored according to the American Academy of Sleep Medicine (AASM) criteria, Version 2.6 (2020) [25].

### 2.5 Anthropometric Assessment

Anthropometric data were collected as recommended by the World Health Organization [26].

### 2.6 Vascular and Hemodynamic Parameters and Arterial Stiffness Indices

An oscillometric 24‐h ambulatory blood pressure monitoring device (Mobil‐O‐Graph; IEM, Germany) was used to assess hemodynamic and vascular parameters and arterial stiffness indices [18] validated by the American Heart Association's Council on Hypertension and The British Hypertension Society [27, 28]. This device utilizes the ARCSolver algorithm (ARCSolver method, Austrian Institute of Technology) to estimate arterial stiffness by providing variables such as pulse wave velocity (PWV) and the Augmentation Index adjusted for a heart rate of 75 beats per minute (AIx@75). PWV was determined using a mathematical model based on pulse wave parameters and wave separation analysis [29]. AIx@75 was calculated from the difference between the peak of the reflection wave (P2) and the peak of the incident wave (P1) and was expressed as a percentage of the central pulse pressure (cPP), corrected for a heart rate of 75 bpm [AIx@75 = 100 × (P2 ‐ P1)/cPP] [30]. The monitor also provided the measurements of Heart Rate (HR), Systolic (SBP), and Diastolic (DBP) Blood Pressure, and Pulse Pressure (PP), centrally and peripherally. These factors have been recently proposed to estimate cardiovascular risk in many populations [13, 31].

To ensure reliable data, participants were instructed to remain seated with their feet and arms supported. The examination was conducted after 10 min of rest [32]. Three consecutive measurements of all parameters were taken, and the mean value, if acceptable, was used for analysis. Participants were also instructed to abstain from stimulating foods (such as coffee, cola‐based soft drinks, and chocolate), alcoholic beverages, and nicotine, as well as to avoid physical activity for at least 24 h before the exam [33].

### 2.7 Statistical Analysis

The statistical analysis followed the recommendations of the SAMPL (Statistical Analysis and Methods in the Published Literature) guidelines to ensure transparency and reporting accuracy. Continuous variables were expressed as mean ± SD (standard deviation) or median (Interquartile Range, IQR). Data normality was assessed using the Shapiro–Wilk test. Categorical variables were described as percentages. Correlations between different variables studied were performed using the Pearson's or Spearman's correlation coefficient, when indicated.

Multivariate regression analysis was performed. This approach enabled adjustment for potential confounders, including age, BMI, hypertension, diabetes, and medication use. Effect sizes (correlation coefficients and regression coefficients) are reported alongside their respective 95% confidence intervals (95% CI) to convey the precision of the estimates. The analysis was developed using the GraphPad Prism program (version 5.0; GraphPad Software Inc., La Jolla, California). The significance level adopted was 5% (*p* < 0.05). All hypothesis tests were two‐sided.

## 3 Results

The study population had a mean BMI within the obesity range. There was a high prevalence of cardiometabolic conditions, particularly hypertension, dyslipidemia, and diabetes. The medication profile reflected this clinical scenario, with frequent use of RAAS blockers, oral antidiabetic agents, and statins (Table 1).

**Table 1:** Sample characterization and medications.

Variables	Mean (SD)
Age (years)	62.07 (8.8)
Height (meter)	1.65 (0.07)
Weight (kg)	84.06 (12.6)
BMI (Kg/m^2^)	30.13 (3.7)
*Gender*	*N* (%)
Male	33 (55)
*Comorbidities*	*N* (%)
Systemic arterial hypertension	30 (50.0)
Diabetes	19 (31.6)
Dyslipidemia	24 (40.0)
*Medication*	*N* (%)
AT1 or ACE blockers	22 (36.7)
Calcium channel blockers	8 (13.3)
Beta blockers	9 (15.0)
Statins	14 (23.4)
Metformin	18 (30.0)
Insulin	3 (5.0)
SGLT2 inhibitors	6 (10.0)
Others drugs	9 (15.0)

*Note:* The medications listed refer to the treatment of hypertension, diabetes, and dyslipidemia only.

Table 2 displays the median and mean values of the main polysomnographic variables measured in the participants. Nineteen participants were categorized as having moderate and 41 severe OSA based on an AHI [2]. The respiratory assessment revealed a pattern of severe sleep‐disordered breathing, with evidence of sustained nocturnal hypoxemia and impaired oxygenation stability throughout the night. The median and mean percentage durations of all sleep stages are also presented in Table 2.

**Table 2:** Polysomnographic variables of the participants.

Variables	*N* a	Median (IQR)	Mean ± SD
AHI (events/h)	60	35.6 (26.4–48.4)	38.7 ± 14.1
Sleep efficiency (%)	57	86 (80.3–93.8)	82.10 ± 20.7
REM sleep latency (min)	57	99.25 (87.5–189.0)	136.9 ± 76.9
Mean SpO_2_ (%)	60	94.0 (92.0–95.0)	93.36 ± 2.1
Minimum SpO_2_ (%)	60	80.0 (73.0–86.0)	78.88 ± 8.1
T/SpO_2_ < 90% (%)	60	5.4 (2.1–14.8)	13.42 ± 17.8
% N1 sleep stage	57	2.6 (2.0–4.35)	7.45 ± 8.7
% N2 sleep stage	57	54.5 (12.6–61.5)	60.53 ± 14.0
% N3 sleep stage	57	11.7 (3.0–12.7)	13.75 ± 9.0
%REM sleep stage	57	12.25 (6.0–17.3)	16.76 ± 7.2

Abbreviations: AHI, Apnea‐Hypopnea Index; REM, Rapid Eye Movement; SpO_2_, Peripheral Capillary Oxygen Saturation. T/SpO₂ < 90%, the duration or percentage of total recording time during which the SpO₂ was below 90%.

^a^ Some variables contain missing data, as certain polysomnographic measurements were not recorded in the medical charts of three participants.

Table 3 reveals vascular and hemodynamic parameters and arterial stiffness indices evaluated by the Mobil‐O‐Graph device.

**Table 3:** Peripheral and central blood pressure values, hemodynamic variables, and arterial stiffness indices.

Variables	Value
*Peripheral blood pressure (mmHg)*	
Systolic blood pressure (pSBP)	128.0 ± 12.87
Diastolic blood pressure (pDBP)	83.0 ± 10.33
Mean arterial pressure (MAP)	103.60 ± 10.10
Pulse pressure (pPP)	44.98 ± 11.10
*Central blood pressure (mmHg)*	
Systolic blood pressure (cSBP)	118.20 ± 11.23
Diastolic blood pressure (cDBP)	84.38 ± 10.47
Pulse pressure (cPP)	33.85 ± 8.97
*Hemodynamics parameters*	
Systolic volume (mL)	68.29 ± 11.75
Cardiac output (L/min)	4.86 ± 0.51
Total vascular resistance (s*mmHg/mL)	1.31 ± 0.33
Cardiac index (L/min/m^2^)	2.51 ± 0.32
Heart rate (bpm)	73.03 ± 12.1
*Arterial stiffness*	
Augmentation pressure (mmHg)	8.49 ± 5.06
Coefficient of reflection	64.90 ± 8.26
AIx@75 (%)	22.6 ± 10.33
Pulse wave velocity (m/s)	9.06 ± 1.43
Pulse pressure amplification	1.34 ± 0.18

*Note:* Data presented as mean ± standard deviation.

Abbreviation: AIx@75, augmentation index adjusted to a heart rate of 75 bpm.

**Figure 1:**
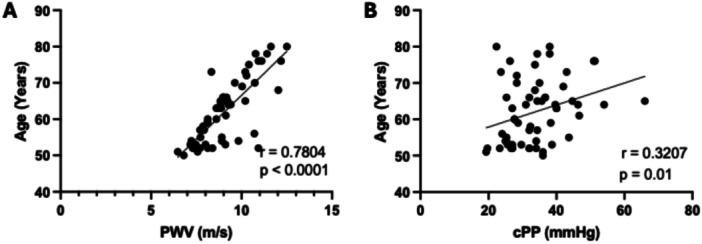
Scatter plots to demonstrate the correlation between Pulse Wave Velocity (PWV) (A) and cPP (B) with age. (A) Scatter plot showing the relationship between pulse wave velocity (PWV) and age. (B) Scatter plot showing the relationship between cPP and age. Spearman correlation coefficient test.

A significant positive relationship was found between Age and PWV (*r* = 0.7804; CI95% = 0.6508 to 0.8658; *p* < 0.0001) (Figure 1A). The correlation coefficient for age and cPP was also statistically significant (*r* = 0.3207; CI95% = 0.06933 to 0.5432; *p* = 0.01) (Figure 1B).

**Figure 2:**
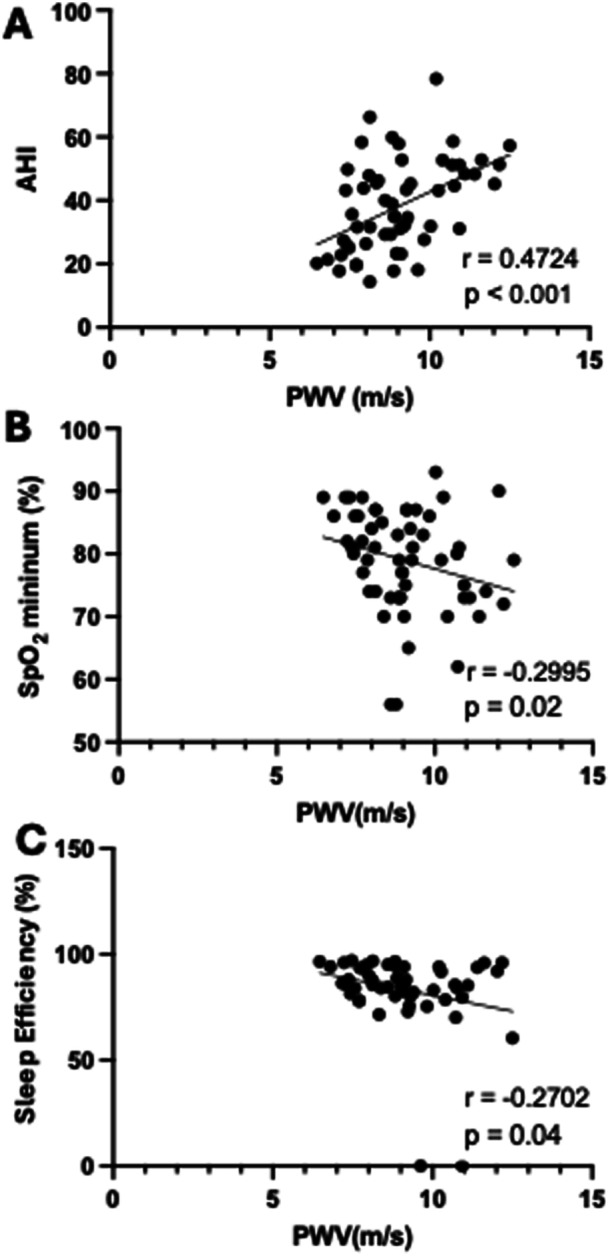
Scatter plots to demonstrate the correlation between Apnea and Hypopnea Index (AHI), SpO_2_ minimum, and Sleep efficiency with Pulse Wave Velocity (PWV). (A) Scatter plot showing the relationship between Pulse Wave Velocity (PWV) and Apnea‐Hypopnea Index (AHI). (B) Scatter plot showing the relationship between PWV and minimum SpO_2_. (C) Scatter plot showing the relationship between PWV and Sleep efficiency. Spearman correlation coefficient test.

The main arterial stiffness index in the adult population, PWV, showed a statistically significant relationship with important OSA severity markers. A positive relationship was seen for PWV and AHI (*r* = 0.4724; 95% CI = 0.2438 to 0.6512; *p* < 0.001;) (Figure 2A). On the other hand, PWV was inversely related to minimum SpO_2_ (*r* = – 0.2995; 95% CI = –0.5234 to –0.03684; *p* = 0.02) (Figure 2B). Sleep efficiency, defined as the ratio of the total time spent asleep to the total time spent in bed (expressed as a percentage), was inversely related to PWV (*r* = –0.2702; CI95% = –0.4999 to –0.004971; *p* = 0.04) (Figure 2C).

In the unadjusted model (Table 4), the AHI, sleep efficiency, and Minimum SpO₂ were significantly associated with PWV. Higher AHI values were correlated with increased arterial stiffness, while lower sleep efficiency was associated with higher PWV. In addition, lower minimum oxygen saturation (SpO₂) was also significantly related to higher PWV, indicating that nocturnal hypoxemia may contribute to increased arterial stiffness.

**Table 4:** Linear regression models evaluating the association between sleep parameters and pulse wave velocity (PWV).

Predictors	Unadjusted model Beta (95% CI)	*p*	Adjusted model Beta (95% CI)	*p*
AHI (events/h)	0.036 (0.014 to 0.058)	**0.002**	0.010 (–0.007 to 0.026)	0.25
Sleep efficiency (%)	–0.021 (–0.036 to –0.005)	**0.009**	–0.015 (–0.026 to –0.005)	**0.005**
Minimum SpO₂ (%)	–0.042 (–0.082 to −0.002)	**0.04**	0.044 (–0.066 to 0.154)	0.43
Age (years)	—	—	0.112 (0.085 to 0.139)	**< 0.001**

*Note:* Dependent variable: Pulse Wave Velocity (m/s). Model adjusted for age, sex, body mass index, hypertension, diabetes, and medication use (β‐blockers and ACE/AT1 receptor antagonists). All VIF < 2.0, indicating absence of multicollinearity.

After adjustment for potential confounders, including age, sex, body mass index (BMI), hypertension, diabetes, and the use of β‐blockers or ACE/AT1 receptor antagonists, the associations between sleep parameters and PWV were attenuated. Only age remained an independent predictor of arterial stiffness (Beta = 0.112; 95% CI: 0.085 to 0.139; *p* < 0.001).

Sequential models adjusted first for age, then for sex and BMI, revealed a progressive loss of significance for AHI and sleep efficiency, confirming that the initial associations were largely explained by age‐related vascular changes. The inclusion of hypertension, diabetes, and medication use did not modify the significance levels or improve model fit. Variance inflation factors were consistently below 2.0, ruling out multicollinearity.

## 4 Discussion

This study showed associations of PWV, considered the gold standard for arterial stiffness assessment in clinical practice, with the main polysomnographic parameters used to assess OSA severity.

Theorell‐Haglöw et al. (2019) have explored data from 365 patients from six studies performed in a single centre and analyzed the relationship between arterial stiffness, through PWA determined using applanation tonometry, and the severity of OSA [17]. These authors identified a positive association between AHI and AIx@75, which is also an independent marker of arterial stiffness. This work reinforced the importance of evaluating the use of predictors of cardiovascular events such as those related to arterial stiffness in the population suffering from OSA. Our study, in turn, shows an even stronger association of important parameters related to the severity of OSA with the main indicator of arterial stiffness, the aortic PWV [34, 35, 36].

It is well known that individuals with moderate‐to‐severe OSA have a higher risk of cardiovascular problems such as hypertension, heart failure, stroke, and myocardial infarction, all of which can contribute to increased mortality rates, while mild OSA generally does not exhibit the same level of risk [37, 38]. We included in our study only patients with moderate to severe OSA who were not undergoing any treatment for the condition.

Although AHI is the key polysomnographic variable in the classification of OSA, saturation values (Mean SpO_2_, Minimum SpO_2_, and T/SpO_2_ < 90%) are also very important to assess the severity of this condition and also serve as an important guide for treatment [1, 39]. Therefore, we tested the associations between PWV and these indices that refer to the severity of OSA. AHI showed a strong positive association with PWV, suggesting that OSA influences arterial stiffness. In addition, we showed that the lower the minimum saturation (nighttime saturation nadir), the greater the arterial stiffness assessed by PWV (inverse association). These data corroborate the findings of Theorell‐Haglöw et al. (2019) [17]. In our study, both T/SpO_2_ < 90% and Mean SpO_2_ did not significantly associate with PWV (data not shown). Those authors, on the other hand, found an inverse association between AIx@75 and T/SpO_2_ < 90% [17]. Another study by Chung et al. also showed an inverse correlation between this variable and carotid‐femoral PWV [39]. These authors highlight the relevance of nocturnal hypoxemia as a determinant of OSA severity and propose that desaturation‐related indices may provide complementary information beyond AHI. Persistent nocturnal hypoxemia has been linked to structural and functional vascular alterations, particularly in the aorta [40, 41, 42], supporting the rationale for examining its association with arterial stiffness.

An interesting association observed in our study was an inverse association between the sleep efficiency and PWV. Sleep efficiency is a polysomnographic metric that assesses the proportion of time a person spends in bed actually sleeping, compared with the total time spent in bed [2]. Many studies have already shown the importance of the quality of sleeping and total time of daily sleep for cardiovascular health, mainly based on assessment by sleep quality questionnaires [43]. However, more objective assessments that investigate the association between cardiovascular risk markers and PSG parameters inferring sleep quality, are needed. Laharnar et al. (2020) observed that insomniac patients with sleep efficiency below 80% had greater arterial stiffness assessed by pulse propagation time than insomniac patients with better sleep efficiency [44]. We found no other study that accessed polysomnographic sleep efficiency and directly correlated it with predictors of cardiovascular risk. However, sleep efficiency, despite being a good indicator of sleep quality, should be considered together with other aspects of sleep to obtain a more accurate assessment of sleep quality, such as sleep depth (characterized by sleep phases) and total sleep duration. These other parameters, despite having been evaluated in this study, did not show a significant association with arterial stiffness.

Age is a variable that is directly associated with increased arterial stiffness [16, 35, 45, 46, 47, 48]. As expected, in our study, we observed that age had a strong association with PWV. Furthermore, we also found a positive association between age and cPP, which is also considered an index of arterial stiffness and is important in assessing cardiovascular risk [47, 48]. Although not central to the aims of this study, these associations align with the extensive literature describing the influence of aging on vascular remodeling and on the modulation of hemodynamic parameters. As previous research has demonstrated that anthropometric indices of obesity and fat distribution are important predictors of cardiometabolic risk [49], multivariate regression analysis was performed to adjust for these and other potential confounders. The present analysis indicated that, although sleep efficiency, Minimum SpO₂, and AHI were initially associated with arterial stiffness, these relationships lost significance after controlling for age and other confounding variables. Age was found to be an independent predictor of PWV, consistent with previous evidence that vascular stiffening increases progressively with aging due to structural and functional arterial remodeling [50]. The absence of significant effects from comorbidities and medication use suggests that the observed association between sleep parameters and arterial stiffness is mainly age‐dependent rather than independent.

### 4.1 Study Strengths

All patients enrolled in this study were diagnosed with severe‐to‐moderate OSA through Type I PSG, which is considered the gold standard method for diagnosing OSA. Furthermore, all patients were over 60 years old, which is an age group with a higher cardiovascular risk and a higher risk for OSA. Furthermore, the distribution was balanced in relation to sex.

### 4.2 Study Limitations

The limitation of this study is the restricted number of individuals due to the difficulty in finding non‐treated patients affected by moderate‐to‐severe OSA. For this reason, we could not exclude adequately treated hypertensive, dyslipidemic, and well‐controlled diabetic patients, despite recognizing their potential impact on increased arterial stiffness.

## 5 Conclusion

Our results show associations of the PWV with AHI and minimum SpO_2,_ two important polysomnographic parameters used to assess OSA severity. Sleep efficiency was inversely associated with PWV. These findings point to an increase in arterial stiffness as OSA and sleep quality worsen. These findings provide more insights into the relevance of the impact of sleep disorders on cardiovascular diseases.

## Author Contributions

**Erica Silva:** conceptualization, investigation, data curation, formal analysis, and editing of the original draft. **Jessica Giovana Teixeira de Andrade, Eduardo Rodrigues Ferreira Gomes de Camargos, Olivia Mendonça Nunes, Gabrielle Santos Pontello Neves, Patrícia de Souza Pereira:** data curation, methodological support, and revision of the original draft. **Jose Felippe Pinho da Silva, Maria da Glória Rodrigues‐Machado, Daniel Mendes‐Pinto:** conceptualization, formal analysis, investigation, methodological supporting, project administration, validation, and revision of the original draft. **Bruno Almeida Rezende:** conceptualization, formal analysis, statistical analysis, investigation, methodological support, project administration, supervision, validation, and revision of the original draft. All authors read and approved the final manuscript.

## Funding

The authors received no specific funding for this work.

## Ethics Statement

This study was performed in line with the principles of the Declaration of Helsinki. Approval was granted by the Research Ethics Committee of the Faculty of Medical Sciences of Minas Gerais (approval report n. 5513761).

## Consent

Written informed consent was obtained from all the participants before evaluation.

## Conflicts of Interest

The authors declare no conflicts of interest.

## Transparency Statement

The lead author Bruno Almeida Rezende affirms that this manuscript is an honest, accurate, and transparent account of the study being reported; that no important aspects of the study have been omitted; and that any discrepancies from the study as planned (and, if relevant, registered) have been explained.

## Data Availability

The data that support the findings of this study are available from the corresponding author upon reasonable request.
